# Evolutionary autonomous agents and the nature of apraxia

**DOI:** 10.1186/1475-925X-4-1

**Published:** 2005-01-04

**Authors:** Donald S Borrett, Frank Jin, Hon C Kwan

**Affiliations:** 1 Division of Neurology, Toronto East General Hospital, Toronto, Canada; 2 Department of Physiology, University of Toronto, Toronto, Canada

**Keywords:** evolutionary autonomous agents, apraxia, embodiment, genetic algorithm, dynamical system theory

## Abstract

**Background:**

Evolutionary autonomous agents are robots or robot simulations whose controller is a dynamical neural network and whose evolution occurs autonomously under the guidance of a fitness function without the detailed or explicit direction of an external programmer. They are embodied agents with a simple neural network controller and as such they provide the optimal forum by which sensorimotor interactions in a specified environment can be studied without the computational assumptions inherent in standard neuroscience.

**Methods:**

Evolutionary autonomous agents were evolved that were able to perform identical movements under two different contexts, one which represented an automatic movement and one which had a symbolic context. In an attempt to model the automatic-voluntary dissociation frequently seen in ideomotor apraxia, lesions were introduced into the neural network controllers resulting in a behavioral dissociation with loss of the ability to perform the movement which had a symbolic context and preservation of the simpler, automatic movement.

**Results:**

Analysis of the changes in the hierarchical organization of the networks in the apractic EAAs demonstrated consistent changes in the network dynamics across all agents with loss of longer duration time scales in the network dynamics.

**Conclusion:**

The concepts of determinate motor programs and perceptual representations that are implicit in the present day understanding of ideomotor apraxia are assumptions inherent in the computational understanding of brain function. The strength of the present study using EAAs to model one aspect of ideomotor apraxia is the absence of these assumptions and a grounding of all sensorimotor interactions in an embodied, autonomous agent. The consistency of the hierarchical changes in the network dynamics across all apractic agents demonstrates that this technique is tenable and will be a valuable adjunct to a computational formalism in the understanding of the physical basis of neurological disorders.

## Background

The conceptual framework by which a neurological syndrome such as apraxia is presently explained is based on a computational understanding of brain function. Briefly, this framework assumes the existence of well-defined, determinate perceptual representations and motor programs that interact in a fashion similar to the way that symbols are manipulated in a computer. Recent theoretical and applied work in natural and artificial systems, however, has conspired to shift the emphasis in neuroscience from the computational paradigm to a dynamical understanding of brain function [1-5]. This approach emphasizes that cognition occurs in an embodied agent interacting dynamically with its environment and avoids the assumptions of determinate motor programs and perceptual representations implicit in the computational framework [6,7]. The identification of the dynamical changes in an apractic nervous system may provide a causally mechanistic explanation for the syndrome that may complement the higher-level descriptive explanation of the standard computational approach.

The apraxias constitute a spectrum of movement disorders in which there is impairment in the performance of a skilled, learned movement that cannot be attributed to an elementary motor or sensory deficit. Based of the pioneering work of Liepmann [8], the apraxias have traditionally been divided into ideational, ideomotor and limb-kinetic apraxia. Limb-kinetic apraxia, which is felt by many to not be a true apraxia, manifests as slowness or clumsiness of distal limb movements with preservation of knowledge of the appropriate action to perform. Ideational apraxia is characterized by loss of knowledge of how an object is used or as impairment in the sequencing of constituent movements in a complex movement. Ideomotor apraxia is usually diagnosed on the basis of spatiotemporal errors that occur on transitive gesture tasks requiring demonstration of the pantomime appropriate to specific object use [9]. Asking a patient to demonstrate how they would use a comb or a hammer would be typical transitive gesture tasks used in the assessment of the presence or absence of ideomotor apraxia. In many cases of ideomotor apraxia, the spatiotemporal errors improve when the object is actually used rather than when its use is pantomimed. In addition, the movements are often performed normally when they occur spontaneously but are impaired when the patient is instructed to perform the movement. The patient may scratch his nose spontaneously but may be unable to perform this task to command. This voluntary-automatic dissociation, although frequently seen in ideomotor apraxia, is not universal [10].

The standard conceptualization of apraxia is based on a two-system model of action: a conceptual system, located in the dominant parietal lobe, and a production system localized to the frontal lobe [11]. Dysfunction of the former would lead to ideational apraxia and dysfunction in the latter would result in ideomotor or limb-kinetic apraxia. With improved knowledge of the multiple frontoparietal circuits that subserve visuospatial transformations for reaching, somatosensory transformations for postural adjustments and the coding of peripersonal space for limb and neck movements, it has been possible to analyze the deficits which occur in apraxia in more detail than afforded by the standard conceptualization. Based primarily on primate studies, specialized circuits responsible for more detailed properties of action have been identified and provide a framework by which the idiosyncratic deficits evident in the apractic patient that defy explanation by the simple two-system model have been explained [11].

Regardless of whether the standard two-system scheme of action or the more detailed model based on multiple, specialized parietofrontal circuits is used, the standard conceptualization of the origin of apraxia still conforms to the computational paradigm in which a specific motor program is activated based on the conceptual framework in place in the dominant parietal lobe. Similarly, the usual explanation for the automatic-voluntary dissociation frequently seen in ideomotor apraxia relies on this computational framework. This framework postulates that a verbal command establishes a conceptual bias in the parietal lobe that activates the appropriate motor program in the frontal lobe. In ideomotor apraxia there would be a disconnection between the instruction and the effector mechanisms frontally. To explain the preservation of the corresponding automatic movement, alternative pathways not dependent on the parietal lobe would need to remain functional.

Evolutionary autonomous agents (EAAs) are robots or robot simulations whose controller is a dynamical neural network and whose evolution is guided by a genetic algorithm. They are embodied agents-either software programs living in a virtual environment or true robots that function in a specified environment. These agents function autonomously in their environments with the agents performing such functions as navigation around obstacles, gathering food, seeking prey or mating partners. [12] Their development is guided by evolutionary algorithms which utilizes a fitness function to select the most appropriate agents for propagation. Motor or sensory activity, in particular, evolves autonomously in response to the constraints of the fitness function without the organizational restrictions imposed by the notions of determinate motor programs or perceptual representations. Such agents provide a system in which the organization of motor or perceptual activity can be followed and analyzed. Because its nervous system is limited to a small number of neuron-like elements, the analysis of the network dynamics of these agents is also more tractable. Their primary value for the neurosciences is that they provide simple systems unencumbered by the assumptions inherent in present day neurosciences that can serve as a test-bed for thinking about neural processing and techniques for deciphering these processes [13].

To model the voluntary-automatic dissociation seen in ideomotor apraxia, a lesioned EAA needs to demonstrate a behavioral dissociation between a movement that the agent does automatically and an identical movement that has a symbolic context. An analysis of the change in network dynamics that occurs in the apractic EAA will provide information on the physical basis of the dissociation without the assumptions inherent in a computational formalism. The extrapolation of results in the EAA to human brain function is based on a principle concerning the organization of complex systems that has been emphasized by Herbert Simon. He suggested that the organization of self-organizing complex systems is dependent only on the behavioral characteristics of the system and not the nature of the constituent elements of the system. "My central theme is that complexity takes the form of hierarchy and that hierarchic systems have some common properties independent of their specific content" [14]. Regardless of whether the system consists of 100 billion interacting living cells or a small number of computer generated input-output units, it is not inconceivable that the organizational structure of the systems will be similar if their demands are identical and if they are allowed to evolve autonomously. In the absence of an evolved agent with language capabilities, a paradigm is needed that captures the essential elements of an inability to move to command with preservation of that same movement if performed spontaneously. It is felt that the ability to move to a target location without ongoing visual feedback from the target represents the simplest activity upon which the dynamics and connectivity of a robotic system could develop cognitive functions such as off-line reasoning [15]. Since predicative activity such as language may originate in this type of network activity, this particular movement paradigm may be used as a surrogate for a verbal command.

The presence of multiple time scales in the dynamics of a neural network is indicative of a temporal hierarchical structure. Simon discussed the presence of higher and lower frequency dynamics in complex systems and associated more executive function with the lower frequency components. He stated that "it is generally believed that the relevant planning horizon of executives is longer, the higher their location in the organizational hierarchy" and that "the average interval between interactions are greater at higher than lower levels [14]." In the nervous system, it is also expected that such a multiple time scale framework would occur with a hierarchical structure requiring more executive function reflecting lower frequency dynamics evolving as tasks become more complicated. In this paper, the temporal hierarchical structure of the dynamics of the neural network controller of an EAA will be assessed by the analysis of the power spectral distribution and Hurst exponent of all nodes in the network [16]. The analysis will be applied to an EAA model of the voluntary-automatic dissociation seen in ideomotor apraxia in an attempt to causally explain its physical origin.

## Methods

A simulation platform, WEBOTS (Cyberbotics, Switzerland), was used to simulate the movement of a Khepera robot (K-TEAM Corporation, Switzerland) in a dark 1 m square arena without walls or obstacles. The Khepera is a two-wheeled mobile robot with eight ambient light sensors and a rotation encoder for each wheel. The Khepera also has eight infrared proximity sensors but these were not activated in the present study. The neural network controller was composed of a layer of 5 fully connected radial basis function units or neurons. In addition, each unit projected to the two linear units or motor neurons and received inputs from the eight light sensors and the two wheel encoders.

A combined genetic and adaptation algorithm was used similar to that described by Urzelai and Floreano [17]. Briefly, the first generation at the beginning of the evolution was composed of a hundred individuals with randomly assigned synaptic weights and adaptation rules. A chromosome was made up of all the synaptic weights and their associated adaptation rules. A standard genetic algorithm with cross over and mutation operators were used with the best twenty individuals selected. The roulette wheel selection method, wherein the probability of an individual being selected for a new generation is the individual's fitness as a fraction of the total population fitness, was used. One of five Hebbian adapation rules were associated with each weight, the standard Hebbian, presynaptic, postsynaptic, covariance [17] and no change. Although the synaptic weights were altered by the adaptation rule during the life of each individual, it was the pre-adaptation weights and rules that were transmitted to the next generation. One individual had a lifespan of 75 seconds. Each sensorimotor cycle was 64 ms allowing adaptation to occur over 1172 iterations.

The arena was maintained dark, except at random times when a light of constant intensity would appear at random locations. The robot had to attempt to reach the light. There were two tasks for the robot during each trial. In the first task, the robot simply had to go to the light when it appeared. The light remained on and was extinguished when the robot moved to within 2 body diameters, or 7 cm of the light. After a 1–3 s of random delay (in darkness) another light would appear in the arena at another random location requiring the robot to approach that light. This would continue for a total of 4–6 constant lights. The second task began after this series of constant lights. With the second task the robot had to go to the location of a brief light flash (1 s or 16 sensorimotor cycles in duration) that did not persist while the robot approached the location. Since the robot could not use light intensity to continuously guide its movement to the location, successful completion of this task required the robot to have some notion of objective orientation and distance. Each trial lasted a total of 75 seconds.

The fitness function *F *was defined as:

*F *= *T *(*s*)·*n*



where *T *(*s*) = 1 for *s *≤ 1 and

*T *(*s*) = *s *for s > 1,

*n *the number of lights reached, *d*_*f *_the average distance of the robot from the flash point once the flash occurred, *t*_*f *_the time between flash onset and end of simulation, and *t*_*s *_the duration of simulation. Mutation rate was in the 0.2 – 0.5% range.

Evolution was continued until the fitness plateaued. Three populations were evolved requiring up to 1300 generations before there was a clear plateau. Individuals in each population were screened for their ability to accomplish the tasks. Each screening trial was the same as the trials in which the robots were evolved, that is, a 75 second epoch during which the robot had to approach several sustained light locations followed by a single unsustained light flash. Invariably, individuals appeared first that were able to go to the sustained light but failed to respond to the light flash. Eventually, individuals with high fitness were found that were able to successfully complete both tasks. From this latter group, lesions were introduced into the neural networks and the subsequent individual was screened with the same 75 s trial. Individuals were subsequently identified in whom the lesion resulted in the loss of the ability to move toward the brief light flash with preservation of the ability to move toward the sustained light.

The lesions that were introduced into the networks were inactivations of single synapses. Typically, an individual that could successfully accomplish both tasks was screened for apraxia by lesioning each synapse in the network and observing the subsequent behavior of that individual. Although individual neurons could have been inactivated, this approach was avoided because the network only had 5 neurons and the resulting lesioned individual often demonstrated gross motor deficits.

Once individuals were identified which could successfully accomplish both tasks and in whom a specific lesion resulted in a dissociation between movement towards the sustained light and movement towards the light flash, their network dynamics were characterized in two ways. First, a fast Fourier transform (FFT) was performed on the activation pattern of all 5 neurons in the network. The time window chosen in the calculation of the FFT was the entire test epoch. The Hurst exponent was then calculated by the average wavelet coefficient method [18] for all 5 neurons in the network again using the entire test epoch.

## Results

Five agents were identified which were able to successfully accomplish both tasks and in whom a lesion resulted in the dissociation between the two tasks. Analysis was performed on all 5 of the neurons in each individual. Five trials were used in each individual to allow statistical analysis of the Hurst exponent data.

**Figure 1:**
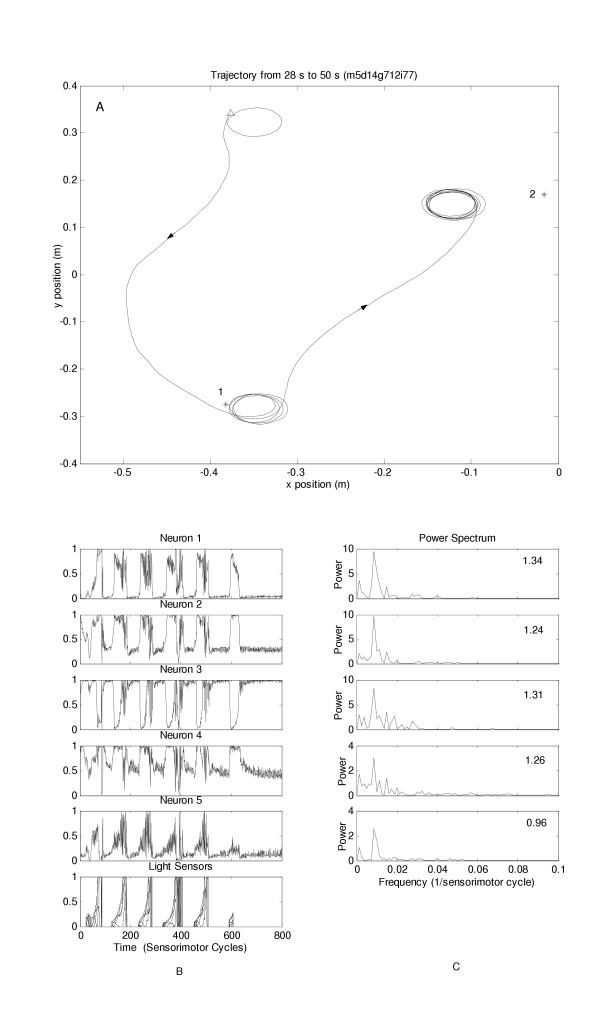
(a) Trajectory of a robot which was able to accomplish both tasks; only the last 3 movements are shown. The robot begins at the open triangle. It then moves to position 1 (sustained light) and then to position 2 (brief light flash). (b) Activity from each neuron of the network and from the light sensor; data for the entire epoch is shown which constituted movements to 5 different sustained lights followed by 1 brief light flash. (c) FFT data for all five neurons calculated over the entire epoch; the value of the corresponding Hurst exponent for each neuron is on the top right.

**Figure 2:**
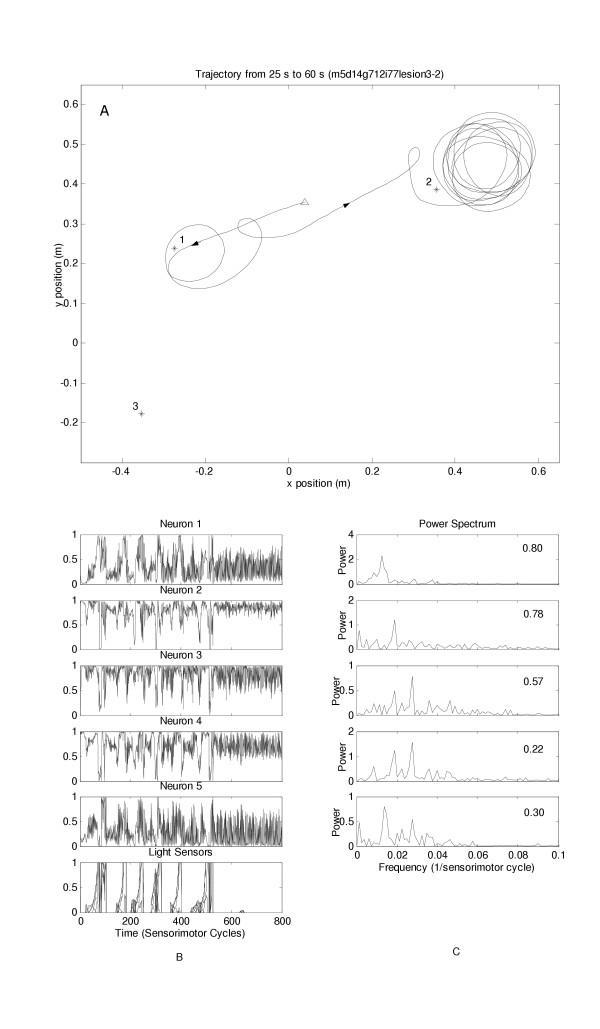
(a) Trajectory of a lesioned robot which lost the ability to move to the brief light flash; only the last 3 successful movements are shown. The robot begins at the open triangle. It then moves to position 1 (sustained light) and then to position 2 (sustained light). The robot remained at position 2 despite the occurrence of a brief light flash at position 3. (b) Activity from each neuron of the lesioned network and from the light sensor; data for the entire epoch is shown which constituted movements to 6 different sustained lights and inability to move to the brief light flash. (c) FFT data for all five neurons of the lesioned robot over the entire epoch. The value of the corresponding Hurst exponent of each neuron is on the top right.

Figures 1 and 2 demonstrate prototypical data obtained for one individual which was able to accomplish both tasks prior to lesioning and demonstrated a dissociation after lesion. In figure 1, before lesioning, only the trajectories to the last constant light stimulus followed by the brief light flash are shown. In figure 2, after lesioning, the last two trajectories to the sustained light stimuli are shown with the agent failing to approach the brief light flash. Individual neuron activity of all 5 neurons for the entire test epoch before and after lesioning along with the FFT data of each neuron before and after lesioning is also shown. The Hurst exponent for the entire epoch for each neuron is also indicated. Visual inspection of the FFT data showed a consistent trend in all neurons with a relative loss of low frequency components in the apractic robots compared to the normal situation.

We computed the Hurst exponents of the 25 neurons from the 5 individuals drawn from 3 populations. Five trials were executed for each individual. When comparing the Hurst exponents for each neuron before and after the lesion, highly significant differences were obtained (p < 0.03 to 10^-7^) for 24 of the 25 neurons, with the remaining neuron showing the same trend but not reaching statistical significance. The grand average of the 125 Hurst exponents (5 trials each for 25 neurons) was1.0957 ± 0.2325 for the normal condition, and 0.8292 ± 0.1833 for the apractic condition.

Both the obvious frequency changes in the FFT data and the Hurst exponent calculations show that the loss of the ability to perform a movement that has a symbolic context is associated with a loss in the longer time scales (low frequency components) in the hierarchical organization of the neural network Since the calculation of the Hurst exponent is based on the full test epoch and since most of the robot movement during this period was the movement to the constant light rather than the light flash, this result also demonstrates that the strategy for both movements was influenced by the lesion despite maintained ability to move to the constant light.

## Discussion

Despite significant advances in the understanding of the anatomy and physiology of normal movement and the pathophysiology of movement disorders, their conceptual framework still relies on the computational paradigm used by cognitive science and neuroscience for decades. This approach assumes the existence of determinate structures in the CNS that are manipulated in the same fashion that a computer manipulates symbols or language combines words. In the last 10 years, there has been a shift in focus with appreciation of the limitations of the computational approach and the realization of the importance of embodiment in conceptualization of motor behavior. When the social and the environmental influences on behavior are analyzed without prejudice, it becomes clear that indeterminacy is a hallmark of our functioning [19] and the idea of determinate perceptual representations and motor programs is an assumption commensurate with the approach of the natural sciences in general. This indeterminacy is captured in the formalism of dynamical systems theory. EAAs provide a system by which development of cognitive structures evolve in an embodied agent constrained only by the nature of the environment and the definition of fitness. They assume no structural characteristics of perception or movement but provide a forum by which sensorimotor structures arise as the organism self organizes in response to its interaction with the environment. Because they possess a simple network controller, the analysis of their development and architecture is more tractable than a similar analysis of the human nervous system.

We have demonstrated that in an evolved agent with two modes of environmental interaction that can be viewed as automatic and symbolic, lesions that produce the equivalent of the automatic-voluntary dissociation seen in ideomotor apraxia cause a change in the hierarchical organization of its dynamical neural network controller with a loss of the longer duration time scales corresponding to the loss of symbolic function. Two techniques were utilized to demonstrate these changes in the network dynamics, a Fourier analysis of the activity of all 5 neurons in the network and the calculation of the their Hurst exponents. With Fourier analysis, EAAs that were able to successfully perform both tasks demonstrated a 1/f power spectral distribution. This type of scale free distribution has been encountered in a number of physical and biological systems including heart rate variability, electrical currents and reaction times in human cognition [16,20]. The 1/f distribution has also been associated with self-organized criticality, a theory of the internal interactions of large systems that has been postulated to govern such diverse natural phenomena as the size of avalanches and earthquakes [21]. This 1/f pattern was altered in the apractic EAAs indicating that this abnormal behavior was associated with a loss of the lower frequency components in the network dynamics. The calculation of the Hurst exponent is based on long range correlations over all time scales in the signal of interest and is a measure of persistence or memory, that is, how long a given fluctuation in a time series will be reflected in future values of the series [18]. The larger the value of the exponent, the more dominant are low frequencies in the time signal. EAAs that could successfully perform both tasks had a larger value of the Hurst exponent compared to the lesioned, apractic agents again indicating the loss of low frequency components in the network dynamics of the apractic agents.

Does the EAA paradigm described capture the essential features necessary to causally explain the automatic-voluntary dissociation seen in ideomotor apraxia? Ideally, to most reliably model this phenomenon, an EAA would need to have language capabilities. Barring that, it is argued that the ability to move to a target site without the continued visual presence of that target captures the minimal requirements necessary to extrapolate to the case of a verbal command. Although controversy exists concerning the conceptual or operational definition of internal representations in evolved agents [22,23], Clark has argued that the notion of representation remains valuable in evolved robotic systems and can be defined well enough to discuss the physical basis of cognition [15]. He distinguished between what he called weak and strong representations. A weak representation in a robotic system is the dynamics and connectivity of the network which is associated with a behavior requiring ongoing sensory feedback. A movement that is done spontaneously and automatically would be a type of behavior whose underlying network would use a weak representation. A strong representation in a robotic system is the network dynamics and connectivity associated with a behavior that does not require ongoing sensory feedback for its successful execution. He argued that the kind of network organization that supports movement in the absence of visual feedback, such as the movement of an EAA to a target flash location, is the simplest kind of strong representation and is prototypical of representations in robotic systems that could support more sophisticated cognitive processes including off-line reasoning. Since language capability would require the presence of strong representations, it was felt that the movement of an EAA to a target location without continued visual presence of the target was an acceptable surrogate for a verbal command.

It was clear while individual EAAs were being observed as they performed the trial in the normal and apractic conditions that the apractic robots often demonstrated minor deficits even in the case in which they were approaching the sustained light target. Usually, this took the form of a mild slowness in movement although other deficits were seen such as altered trajectories toward the sustained light source. It has been assumed that patients with ideomotor apraxia function normally in the environments to which they are accustomed and in which automatic behavior is expected. This has also been suggested as a reason why apraxia is not detected as frequently as its true incidence predicts [10]. This clear cut dissociation would also be predicted by a computational formalism since distinct and determinate motor programs would lend themselves to inactivation of one with maintenance of the other. Recently, this idea has been disputed and, in fact, careful examination of apractic patients reveals deficits even in movements that are performed automatically [10]. The results in our EAA simulation corroborates this more recent analysis. Since determinate motor programs or perceptual representation do not exist in the EAAs, it would be expected that a lesion in the network would have some effect on all aspects of the agent's motor performance.

The notion that a lesion that causes apraxia has a global effect on the patient that is not restricted to those functions that can be considered symbolic was elaborated in detail by Merleau-Ponty [19]. He performed an extensive existential analysis of one patient, Schneider by name, who had suffered a shell injury to his brain. Merleau-Ponty suggested that the standard empirical or cognitive explanations of the nature of apraxia are limited because they fail to take into consideration the phenomenology of apraxia. According to Merleau-Ponty, "Beneath the intelligence as an anonymous function or as a categorical process, a personal core has to be recognized, which is the patient's being, his power of existing. It is here that illness has its seat". He argued that it is "ridiculous to think that the shell splinter directly struck symbolic consciousness" but rather "any pathological degeneration should affect the whole of consciousness." This phenomenological insight was mirrored in the observation that lesions that resulted in a behavioral dissociation in the EAAs had an influence on the dynamics of the network even in the tasks that were behaviorly unchanged. By avoiding the assumptions inherent in the computational framework and by evolving hierarchical self-organization, including symbolic thought, from the most basic level of sensorimotor interactions in an embodied agent, EAAs provide the optimal framework by which these phenomenological observations on the nature of apraxia can be modeled. In fact, taking phenomenological accuracy in addition to physiological plausibility as a constraint in the selection of those EAAs that successfully perform a behavioral task will help to discover those networks whose architecture and function most closely parallel those of the human nervous system [24].

## Conclusion

In an embodied agent with two modes of environmental interaction which can be viewed as automatic and symbolic, lesions that produce the experimental equivalent of voluntary-automatic dissociation of ideomotor apraxia cause a change in the hierarchical organization of its dynamical neural network controller with a loss of the longer duration time scales corresponding to the loss of symbolic function. The identification of the dynamical changes in an apractic nervous system may provide a causally mechanistic explanation for the syndrome that may complement the higher-order descriptive explanation of the standard computational approach. The methodology of EAAs is just beginning to be recognized and it is hoped that further studies can be performed with this methodology to further understand the physical origin of normal and pathological brain function.

This study was funded by a research grant from the Toronto East General Research Foundation.

We thank Weyland Cheng and Owen Wong for assistance in the research.

## Author's contributions

DSB conceived the study, participated in its design and the data analysis and drafted the manuscript. FJ carried out all computer simulations, participated in the study design and the data analysis and contributed to the manuscript draft. HCK conceived the study, participated in its design and the data analysis and contributed to the manuscript draft. All authors read and approved the final manuscript.
